# Process Controlled Ruthenium on 2D Engineered V‐MXene via Atomic Layer Deposition for Human Healthcare Monitoring

**DOI:** 10.1002/advs.202206355

**Published:** 2023-02-22

**Authors:** Debananda Mohapatra, Yujin Shin, Mohd Zahid Ansari, Youn‐Hye Kim, Ye Jin Park, Taehoon Cheon, Haekyoung Kim, Jung Woo Lee, Soo‐Hyun Kim

**Affiliations:** ^1^ School of Materials Science and Engineering Yeungnam University Gyeongsan Gyeongbuk 38541 Republic of Korea; ^2^ Department of Materials Science and Engineering Pusan National University Geumjeong‐gu Busan 46241 Republic of Korea; ^3^ Center for Core Research Facilities Daegu Gyeongbuk Institute of Science & Technology (DGIST) Sang‐ri, Hyeonpung‐myeon Dalseong‐gun Daegu 711‐873 Republic of Korea; ^4^ Graduate School of Semiconductor Materials and Devices Engineering Ulsan National Institute of Science and Technology (UNIST) Ulju‐gun Ulsan 44919 Republic of Korea

**Keywords:** atomic layer deposition, healthcare monitoring, human–machine interface, precious metals, V_2_CT*_X_* MXene

## Abstract

In searching for unique and unexplored 2D materials, the authors try to investigate for the very first time the use of delaminated V‐MXene coupled with precious metal ruthenium (Ru) through atomic layer deposition (ALD) for various contact and noncontact mode of real‐time temperature sensing applications at the human–machine interface. The novel delaminated V‐MXene (DM‐V_2_CT*_x_*) engineered ruthenium‐ALD (Ru‐ALD) temperature sensor demonstrates a competitive sensing performance of 1.11% °C^−1^ as of only V‐MXene of 0.42% °C^−1^. A nearly threefold increase in sensing and reversibility performance linked to the highly ordered few‐layered V‐MXene and selective, well‐controlled Ru atomic doping by ALD for the successful formation of Ru@DM‐V_2_CT*_X_* heterostructure. The advanced heterostructure formation, the mechanism, and the role of Ru have been comprehensively investigated by ultra‐high‐resolution transmission/scanning transmission electron microscopies coupled with next‐generation spherical aberration correction technology and fast, accurate elemental mapping quantifications, also by ultraviolet photoelectron spectroscopy. To the knowledge, this work is the first to use the novel, optimally processed V‐MXene over conventionally used Ti‐MXene and its surface‐internal structure engineering by Ru‐ALD process‐based temperature‐sensing devices function and operational demonstrations. The current work could potentially motivate the development of multifunctional, future, next‐generation, safe, personal healthcare electronic devices by the industrially scalable ALD technique.

## Graphical abstract

Exploring the unique 2D layered materials, delaminated V‐MXene empowered by precious metal ruthenium (Ru) engineering through atomic layer deposition (ALD) techniques in real‐time human–machine sensing interface devices. The precisely controlled incorporation of precious metal Ru by ALD and successful formation of ALD‐Ru@V‐MXene advanced heterostructure demonstrates the ideal sensing performance device, underscoring the importance of next‐generation safe, wearable healthcare soft electronics.

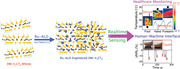

## 1 Introduction

MXene is a 2D carbide/nitrides/carbonitrides, a broad class and variety of inorganic nanomaterials with the general formula M*_n_*_+1_X*_n_* T*_x_*, where M is the early transitional materials and X can be carbon/nitrogen.^[^ ^1^ ^]^ The parent MAX phase is etched with HF or LiF/HCl to get their corresponding MXene phase with functional termination groups like —F or —Cl, —OH, —O due to the processing of wet chemistry materials. This fascinating large 2D family of MXene fields continues to grow due to their desired optical, electrical, mechanical, electrochemical, intrinsic hydrophilicity, ease of processing, and scalability with easy control and tunability of its stoichiometry.^[^ ^2^ ^]^ In addition, these materials intrinsically have oxides surface layers along with good electrical and ionic conductivity due to the central carbide core and tunable surface termination group. A fast redox reaction can be expected from the transition metal oxide surfaces for fast‐charging devices such as pseudocapacitors with high energy density. Among various MXenes practically available, Ti_3_C_2_T*_x_*/Ti_2_CT*_x_* (Ti‐MXene) has been extensively investigated for the applications like energy storage, catalysis, and environmental remediation.^[^ ^2^, ^3^ ^]^ Other than these MXenes, there are ample opportunities for less investigated MXene, like vanadium‐based MXene (V_2_CT*_x_*, V‐MXene).

V‐MXene could be particularly interesting due to its lightweight and variable oxidation state among all other MXene families. The V_2_AlC MAX (V‐MAX) phase has somewhat less orderly powder; hence the V_2_CT*_x_* MXene (V‐MXene) causes some secondary phase of vanadium due to their variable oxidation stage. In the particular interest of V‐MXene, these structures have a large active area per mass. In other words, V‐MXene has only three layers, that is, V_2_C for layer, whereas, the most investigated Ti_3_C_2_T*_x_* MXene has five layers, that is, Ti_3_C_2_ for layer, causing a lower number of atomic layers in the V_2_CT*_x_* MXene over Ti_3_C_2_T*_x_*. Hence the V‐MXene is lighter with a more chemically active vanadium transitional metal with variable oxidation states, which is specifically of interest in electrochemical energy storage, conversion, sensing, and electronic fields.^[^ ^3^, ^4^ ^]^ The few‐layered delaminated V_2_CT*_x_* (DM‐V_2_CT*_x_*) could demonstrate better overall optoelectronic properties over the multilayers. The density functional theory states that nonterminated V_2_C should show a metallic character.^[^ ^4c^ ^]^ At the same time, the experiments suggest the larger organic molecule intercalated multilayered V_2_CT*_x_* shows semiconducting properties, which could demonstrate negative temperature‐resistivity variation resulting from the negative temperature coefficient of resistance.^[^ ^4^, ^5^ ^]^

The selection of appropriate sensing materials and their sensitivity are the front ends of the information accusation and processing for the modern internet of things. Among all types of sensors, the temperature sensors' prominence has increased tremendously given the COVID‐19 era, especially body temperature sensing, starting from medical diagnosis‐treatment to travel tourism. 2D materials such as graphene and the dichalcogenides family have been well investigated for sensing applications considering their high specific surface area and electrical properties. In contrast, the MXene family allows numerous possibilities to tune their compositional stoichiometry, hence the optoelectronic properties.^[^ ^4c^ ^]^ The large surface area, its intrinsic surface hydrophilicity, and water‐intercalated layered structures could give a humidity monitoring ability. In addition, temperature measurement and sensing are now an essential part of daily life, indispensable for industrial and healthcare applications, especially at the human–machine interface. Over the years, metal, its corresponding stable oxides, and inorganic semiconductors have been used as temperature‐sensing materials. Graphene‐based temperature sensing has been extensively investigated among those materials by controlling their reduction rates.^[^ ^6^ ^]^ Ti‐MXenes have recently been explored for their various flexible electronics for healthcare and personal therapy applications.^[^ ^5^, ^6^, ^7^ ^]^ The thermal and chemical stability of Ti‐MXenes are comparatively unstable in the higher oxidative environment, which converts itself to oxide as TiO_2_. For instance, annealing at 1150 °C in the air for 30 s converted into its oxide form TiO_2_ with carbon sheets.^[^ ^8^ ^]^ At the same time, the electronic properties of Ti‐Mxene are strongly dependent on the synthesis procedure, hence the surface termination groups and the interlayer engineering aspects.^[^ ^7b^ ^]^ However, taking advantage of intrinsic Ti‐MXene's surface hydrophilicity and interfacial solid adhesion properties, Ti‐ MXene‐based flexible smart fabrics were also explored for humidity sensing. A commercially available nonwoven cellulose fiber could be converted to flexible smart electronic devices through optimized MXene coating.^[^ ^7a^ ^]^ It is expected that 2D MXene's large surface area and open interlayered structure could act as H_2_O‐induced swelling or contraction behavior for efficient humidity monitoring.

However, despite the individual merits of MXenes and graphene, also by coupling both of them, considerable sensitivity and durability are not achieved. Both are strongly affected by the synthesis/production process and the involvement of the economics while making post‐synthesis improvements. Later, the poor hydrophilicity and associated production/post‐production cost limit the graphene's sensing operability. At the same time, the V‐MXenes with large active area per mass and lighter layered components as compared to Ti‐MXenes remain an unexplored avenue as long as its real‐time sensing and healthcare application concerns. Besides that, the V‐MXene facilitates ideal electron transport channels resulting in fast charge‐carrier transfer compared to other 2D materials such as graphene, MoS_2_, boron nitride, and MXenes.^[^ ^4^, ^9^ ^]^ An open‐layered structure and abundant surface functional groups within the V‐MXene layers support the fast‐sensitive response's facile and readily accessible path to the electrons.

Further selective surface and layered modification of the V‐MXene by precious metals like ruthenium (Ru) through atomic layer deposition (ALD) could enhance overall electronic properties and electron transport channels throughout its layered structure. ALD is an emerging atomic‐level control technique to deposit the precious or rarest noble metals (Pt, Ir, Os, Pd, Rh, and Ru) as single metal atoms or clusters or a few nanometers uniforms and high‐quality thin films through their self‐limiting growth and precision.^[^ ^10^ ^]^ The selective deposition of Ru on V‐MXene in its atomic or cluster form could significantly alter the surface chemistry and overall electronic properties. Also, the connectivity among the V‐MXene layers facilitates better electrical contact throughout the structure through the metal‐MXene embedded heterostructure. Notably, the atomic Ru enhances the surface activity, hence the sensitivity per atom with significantly less use of the precious metals, considering its scarcity. These Ru single atoms/clusters quickly stabilize themselves over the V‐MXene surface due to V‐MXene's intrinsically rich surface functional groups through chemical bonding during the ruthenium‐ALD (Ru‐ALD) process. Over several Ru‐ALD experimentations and optimizations, we introduce plasma‐free ALD of Ru using a novel tricarbonyl(trimethylenemethane)ruthenium, [Ru(TMM)(CO)_3_] metal‐organic precursor, and O_2_ as a reactant for the V‐MXene's surface selective modification. In this study, we developed a novel Ru‐precursor‐based ALD process and also applied it to the sensible and smart use of the precious Ru‐metal on V‐MXene for the first time for its temperature‐sensing healthcare applications. The evolution of the V‐MXene phase and the presence of Ru on V‐MXene is well evidenced through several advanced electron microscopy techniques and comprehensive sensing performance parameters evaluations.

## 2 Results and Discussions

### 2.1 V‐MAX to V‐MXene Phase Evolution by Diffraction and Microscopic Technique

**Figure 1:**
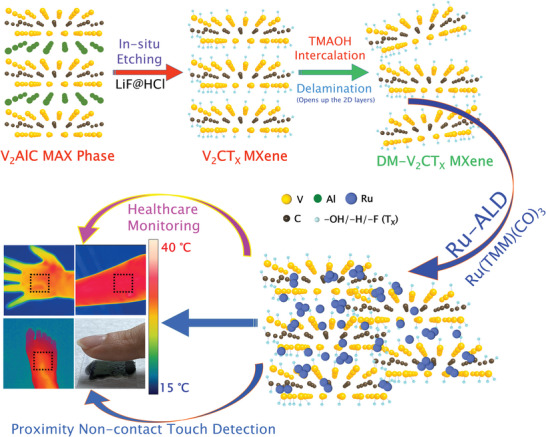
As‐synthesized bulk quantity delaminated V_2_CT*_X_* MXene (DM‐V_2_CT*_X_*) to develop Ru‐ALD Engineered DM‐V_2_CT*_X_* (Ru@DM‐V_2_CT*_X_*) for real‐time skin temperature sensing, noncontact touch, and breathing monitoring.

**Figure 2:**
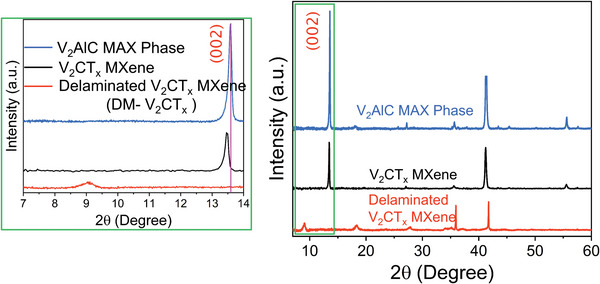
XRD confirmed the phase evolution from the V_2_AlC MAX phase to V_2_CT*_X_* MXene and delaminated V_2_CT*_X_* MXene (DM‐V_2_CT*_X_*).

After selective Al‐etching and forming the V‐MXene phase (**Figure** **1**, detailed in Section 4), X‐ray diffraction (XRD) patterns were recorded to investigate the structural evolution from the V‐MAX phase to the V‐MXene phase and the delamination process. From the XRD spectra of both V‐based MAX and MXene, the change in the intensity ratio of the most characteristic peak (002) can evidently be observed from V‐MAX to the V‐MXene phase. Additionally, the V‐MXene phase (002) peak has been broadened and downshifted to the lower 2*θ* angle. It is typical behavior of etching and exfoliation in the case of layered materials and well‐established in the case of graphene.^[^ ^11^ ^]^ The lower 2*θ* angle locations in **Figure** **2** indicate large organic/inorganic molecules and water molecules as intercalants among the V‐MXene layers. Notably, the insertion of large organic molecules like tetramethylammonium hydroxide (TMAOH) makes them few‐layered, and again, the (002) peak broadened and loss of intensity as compared to the V‐MAX and V‐MXene phases. The characteristic basal plane (002) peak shifted to a lower 2*θ* angle from 13.7° to 13.2° to 9.2° for V‐MAX, V‐MXene, and DM‐V_2_CT*_x_* samples, respectively, with the increasing trend of their d‐spacing explaining the presence of confined intercalants, such as water molecules/ions. The significant (002) peak widening can be due to the reduction in domain size during the wet chemical etching process. The unwanted reactions and some Al‐based impurities caused the appearance of other secondary peaks; however, those are less than the V‐MAX phase. The V‐MAX phase has somewhat less orderly powder; hence the V‐MXene causes some secondary phases of vanadium due to their variable oxidation stage. The shifting of the prominent (002) peaks, some secondary peaks, and differences in the c‐lattice parameter are reported previously in other MXenes to understand the phase evolution from its corresponding MAX phase.^[^ ^3^, ^4^ ^]^ One of the reasons could be the successful intercalation of water molecules among the V‐MXene layers, especially at the lower lateral dimensions. Other reasons may be the remains of unreacted products among those layers, especially during the co‐intercalation of large organic molecules like TMAOH. Comparing the XRD patterns of both V‐MXene and delaminated V‐MXene, most of the V‐MAX phase characteristic peaks disappeared and immensely weakened, after etching and subsequent delamination process, hence, qualified for aimed sensing applications.

The overall morphology of all the products from the V‐MAX phase to V‐MXene and DM‐V_2_CT*_X_* were investigated using Field‐emission scanning electron microscope (FESEM) to distinguish their structural evolution over the synthesis conditions and processes. The morphologies are typical 2D exfoliated graphite‐related materials with compact and layered structures. From the edges of Figure S1A,B, Supporting Information, it is clear that in situ etched V‐MXene powders are composed of multilayered V_2_C MXene layers. In contrast, the later stage with delamination by large intercalant molecules of TMAOH further opens up the layers, as observed in Figure S1C,D, Supporting Information. A typical 2D sheet‐like structure is quite reminiscent of the accordion‐shaped Ti‐MXenes.^[^ ^1^, ^2^, ^4^ ^]^ The expanded accordion‐like V‐MXene structures can be ascribed to the wet‐chemical etching synthesis condition where a large amount of H_2_ gases escaped due to the exothermic nature of in situ hydrofluoric (HF) formation.

### 2.2 Advanced Electron Microscopic Evidence on Ru‐Atomic Layer Deposition Engineered V‐MXene (or DM‐V_2_CT*_X_* MXene)

**Figure 3:**
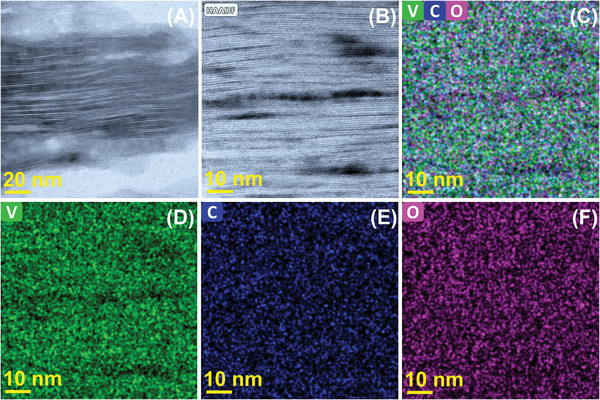
DM‐V_2_CT*_X_* MXene internal microstructure and elemental mapping. A,B) HAADF STEM, C–F) STEM‐EDS elemental mapping images conforming to the V and C.

**Figure 4:**
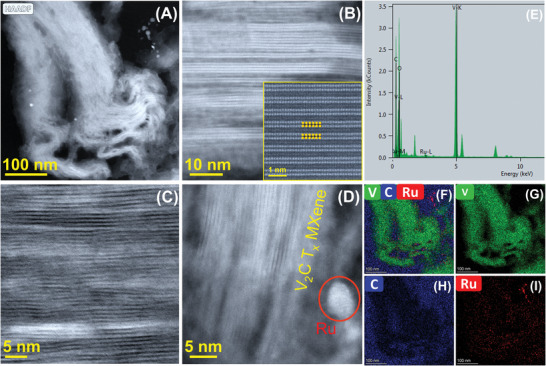
Ru‐ALD engineered DM‐V_2_CT*_X_* MXene microstructure and elemental mapping. A) HAADF STEM showing the presence of layered DM‐V_2_CT*_X_* MXene structure and the distribution of Ru atoms/clusters. B,C) HR‐STEM of the well‐defined layered structure of DM‐V_2_CT*_X_* MXene throughout the sample and inset (B) confirms the opening of V_2_CT*_X_* MXene layers after the removal of Al‐layers. D) HR‐STEM of both layered DM‐V_2_CT*_X_* MXene and Ru lattices. E) Super‐X EDS elemental spectra confirming the elements V, C, Ru, and F–I) their corresponding elemental mapping images. The atoms in (B) inset are shown with the same colors as illustrated in Figure 1 after the etching and delamination process.

To further probe the exact 2D layered stacking, internal microstructure, crystallographic ordering, constituent elements, and stochiometry of V‐MXene were probed by ultra‐high‐resolution scanning transmission electron microscopy (UHR‐STEM) advanced electron microscopy tools equipped with next‐generation spherical aberration correction technology and fast, accurate elemental mapping‐quantification through its super X/dual X energy dispersive X‐ray spectroscopy (EDS) detectors. **Figure** **3**A,B confirms the conversion of 2D layered V‐MXene from the 3D V‐MAX phase. It is also corroborated by **Figure** **4**B and its inset, 4C HR‐STEM images of the well‐defined layered structure of DM‐V_2_CT*_X_* MXene throughout the sample. Hence, wet chemical etching of the V‐MAX phase to the V‐MXene phase has not disrupted the stacking 2D layers, even after the delamination process (DM‐V_2_CT*_X_*). The SAED hexagonal lattice pattern in Figure S2D, Supporting Information, could also be evidence of the maintenance of crystallographic ordering. A TEM and HR‐TEM image in Figure S2A,B, Supporting Information, also corroborates the finding of advanced high‐angle annular dark‐field scanning transmission electron microscopy (HAADF STEM) and HR‐STEM images of its 2D layered nature and well crystallinity (Figure S2C, Supporting Information) even after the etching and delamination process. To estimate the content of V, Al, and C before and after etching and delamination, we performed EDS analysis (Figure S2E, Supporting Information) and its corresponding elemental mappings in Figure S2F–I, Supporting Information. EDS determines the majority of elements contributed by vanadium, carbon, oxygen, and neglected amount of residual/impurities aluminum. EDS elemental mapping images equipped with the HAADF STEM tool conform to the clear presence of major constituent elements V, C, and O in Figure 3C–F. The same has also been observed in Figure S3, Supporting Information, EDS as‐received data with the statistic of each element concentration to confirm the presence of both V and C as major constituents. Due to the lack of a V_2_AlC peak in the above XRD pattern (Figure 2), we can assume all aluminum etched, and some remained as aluminum compounds like aluminum fluoride salts reflected in the EDS spectra in Figures S2E and S3, Supporting Information. The presence of carbon may be overestimated due to contamination; hence the V: C ratio would be nearly 2:1, provided the MXene chemistry doesn't alter as predicted by EDS stochiometry. The oxygen content may arise due to the presence of intercalated H_2_O molecules in MXene layers during wet chemistry synthesis and processing. XRD and EDS measurements complement the etching process of aluminum from the V‐MAX phase and the formation corresponding V‐MXene phase.

The accordion‐like delaminated V‐MXene structure confirmed by FE‐SEM, HAADF STEM results could play an essential role in healthcare sensing applications crediting to their few‐layered structure, which contributes to a large surface area by increasing the electrical contacts. Later, the precise and area‐selected Ru deposition using the sophisticated ALD technique could minimize the face‐to‐face layer stacking and act as 3D metal contacts among those layers for easy electron access during sensing activity (Figure 4). Taking advantage of V‐MXene's semiconducting nature and interlayer swelling after the deamination process, the change in resistance, hence the sensing performance, could arise from free carrier concentration and the facile transport of those free carriers among the V‐MXene layers.^[^ ^4a,c^ ^]^ These phenomena could be further fueled by the selective deposition of precious metals like ruthenium using ALD, possibly doping, which could influence the V‐MXene's surface termination, hence the overall conductivity. ALD allows fabricating of sensor electrodes with highly conformal and uniform coatings with precise control of the amount deposited. This is crucial because thicker active materials might affect the electrode's electrical conductivity and sensing activity unfavorably. In the case of a sensor, which is a surface‐limited process, precise control of deposited materials is essential. Therefore, ALD offers a significant benefit over the processing cost because it reduces the amount of precious metals used and maximizes their utilization.

Moreover, ALD is an effective technique for dispersing active materials onto substrates with large surface areas. ALD is, therefore, a good technique for fabricating electrodes because of its added advantages. These Ru‐ALD engineered V‐MXene surfaces, and internal layers could remain well connected through the selectively deposited Ru metal atoms/clusters to maintain lamellae metal‐MXene heterostructures' stability, hence the internal resistance. Developed Ru‐metal@V‐MXene advanced heterostructure could overcome the individual challenges that either Ru‐metal or V‐MXene has for high‐performance sensing applications. Remarkably, the Ru metallic atom or its clusters could diffuse the extra free electrons to the layered V‐MXene through tunneling, offering an excellent thermal transport channel and improving the overall sensing activity.^[^ ^4c–e^ ^]^ The presence and distribution of Ru atoms/clusters can be observed in Figure 4A without affecting their well‐aligned 2D layered structure of V‐MXene. After chemical etching, the internal gaps among the V‐MXene layers are not disrupted even after the Ru‐ALD engineering process, as shown in Figure 4B,C, and both Ru atoms, V‐MXene in Figure 4D. Importantly, Figure 4B inset HR‐STEM image seemingly shows the removal of Al layers and widened the V‐MXene layers through the delamination process with a sheet thickness of around 0.5 nm. The Super‐X dual EDS proves the presence of Ru along with the V, C, and O. Their corresponding mapping images clearly show the uniform dispersion and spatial distribution of Ru atoms/clusters in Figure 4E and Figure 4F–I, Supporting Information, respectively.

### 2.3 Spectroscopic Evidence on the Role of Ru‐Atomic Layer Deposition on DM‐V_2_CT*_X_* MXene

**Figure 5:**
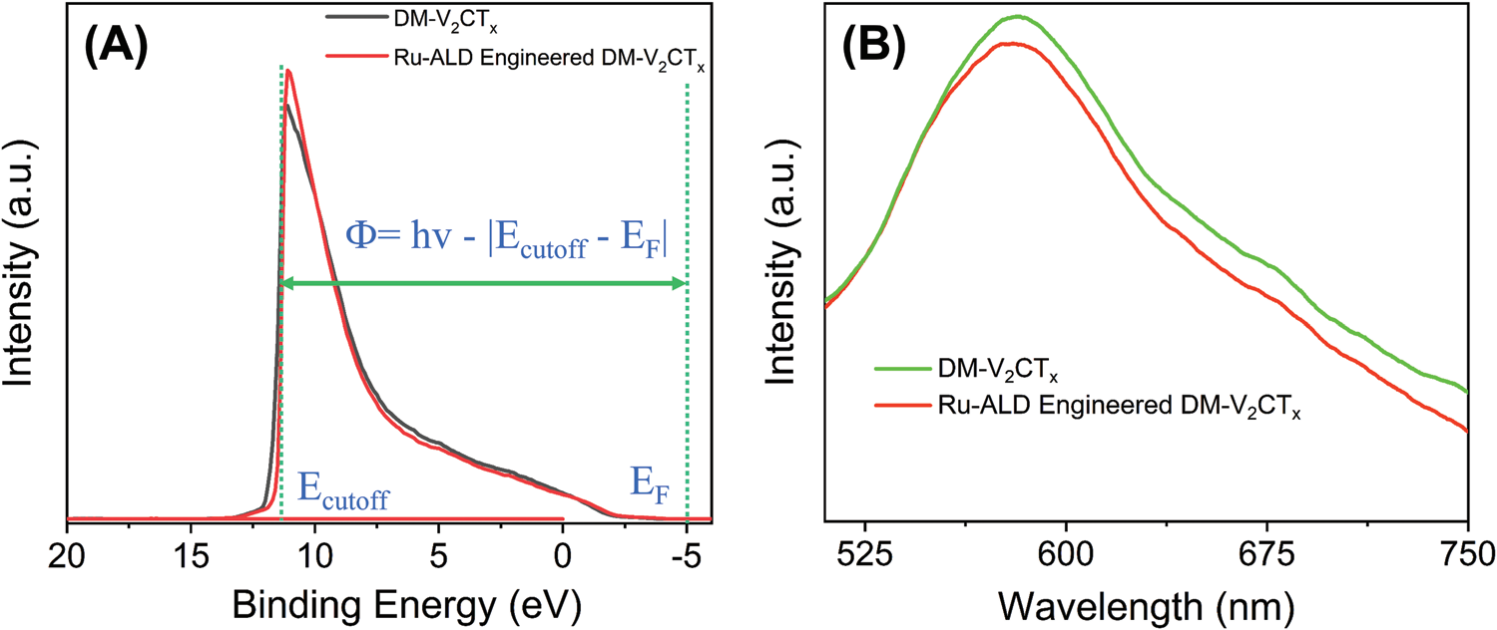
DM‐V_2_CT*_X_* MXene and Ru‐ALD engineered DM‐V_2_CT*_X_* A) He I (21.22 eV) UPS spectrum taken with 5 V bias applied to the sample, corresponding inelastic cutoff (*E*_cutoff_), work function (*ф*), and Fermi edge (*E*_F_) and B) PL spectra.

The semiconducting 2D nanomaterials, such as V‐MXene's work function, are sensitive to any change in Fermi level either by surface engineering or doping.^[^ ^4a–c^ ^]^ It is pertinent to find the role of Ru atoms/clusters on the V‐MXene's surface modification and its resulting overall electronic properties, such as the work function and electron affinity. The sensitive ultraviolet photoemission spectroscopy (UPS) technique (**Figure** **5**A) was employed to understand and evaluate the electronic structure of both V‐MXene and its engineered structure by Ru‐ALD. The calculated work function for Ru‐ALD engineered DM‐V_2_CT*_X_* MXene (Ru@ DM‐V_2_CT*_X_*) is 5.16 eV, which is larger than the DM‐V_2_CT*_X_* MXene, that is, 4.35 eV. This work function difference factor evidently demonstrates the existing electronic structure modification by Ru‐ALD or doping. Higher work function in Ru@DM‐V_2_CT*_X_* potentially indicates the possible role of Ru‐doping in DM‐V_2_CT*_X_* MXene. The increase in work function from 4.35 to 5.16 eV is a clear indication of p‐type semiconductor doping, as discussed in graphene‐like materials.^[^ ^12^ ^]^ This observed difference in work function could easily create the charge transfer between Ru and V‐MXene. Otherwise stated, the availability of electrons near the Fermi level of Ru could effortlessly move to the V‐MXene, facilitating a smooth and uninterruptable charge transfer through Ru@DM‐V_2_CT*_X_* heterostructure during sensing activity. The threefold higher sensing performance and reversibility in the case of Ru@DM‐V_2_CT*_X_*‐based temperature sensor device corroborates the fact in Figure S4, Supporting Information. Hence, the Ru‐metal contact or doping prominently alters the intrinsic V‐MXene's electrical characteristics (Figure S5, Supporting Information), hence their sensing device performance.

To further correlate the optoelectronic properties of both V‐MXene and its Ru‐ALD engineering aspects, photoluminescence (PL) analysis was performed. Interestingly, as discussed above, the carbide core in V‐MXene may have sp^2^/sp^3^ carbon atoms followed by graphitic defects and also other structural defects among the various V‐MXene layers due to the nature of wet chemical synthesis and etching. The defect centers and the interface of Ru@DM‐V_2_CT*_X_* could support the trapping and de‐trapping process.^[^ ^13^ ^]^ Therefore, a minimal modification on the V‐MXene surface internally or externally results in the modification in the electronic states demonstrated by the gradual shifting toward a shorter wavelength or shifting in the peak center. As observed in Figure 5B, Ru@DM‐V_2_CT*_X_* PL spectra are slightly blue‐shifted compared to DM‐V_2_CT*_X_* MXene, demonstrating the role and influence of Ru. The PL intensity is another characteristic of the charge carriers' recombination rate at the trapping and de‐trapping process at scattering centers in layered V‐MXene structures. Ru@DM‐V_2_CT*_X_* MXene PL spectra intensity is lower than the DM‐V_2_CT*_X_* MXene, demonstrating the fact that the overall charge carrier recombinational rate for Ru@DM‐V_2_CT*_X_* is lower than the DM‐V_2_CT*_X_* MXene, hence the role of Ru. When light is irradiated on the Ru@ DM‐V_2_CT*_X_* heterostructure surface, the e^−^‐h^+^ pairs generated at the surface and the recombination rate are delayed due to the presence of Ru, whereas the recombination rate is faster without Ru, causing the PL intensity to be higher as compared to the Ru@ DM‐V_2_CT*_X_* heterostructure.

### 2.4 Temperature Sensing Performance of Ru‐Atomic Layer Deposition Engineered DM‐V_2_CT*_X_* (Ru@DM‐V_2_CT*_X_*)

**Figure 6:**
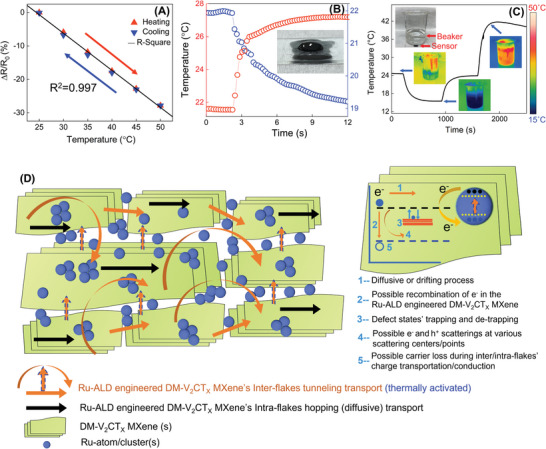
Ru‐ALD engineered DM‐V_2_CT*_X_* MXene real‐time sensing applications and mechanisms. A) Relative resistance changes in the as‐developed temperature sensor as a function of temperature. B) Temperature sensor durability with multiple heating and cooling water‐dropping tests. C) Response to continuous extreme external temperature difference ≈30 to ≈50 °C. D) Conceptual illustrations of possible sensing mechanism and the role of Ru.

As shown in **Figure** **6**, to evaluate the temperature sensor performance of Ru‐ALD engineered DM‐V_2_CT*_X_*, normalized resistance change was utilized as follows:

(1) $$\frac{R-R_{0}}{R_{0}}\times 100\left(\%\right)$$

where *R* is the real‐time measured resistance and *R*_0_ is the initial resistance of the sensor. Additionally, the sensitivity of the resistive temperature sensor is defined as follows:

(2) $$\mathrm{Sensitivity}\;\left[\%\;{}^{\circ }C^{-1}\right]=\frac{R-R_{0}}{R_{0}\times \Delta T}\times 100\;\left(\%\right)$$

where Δ*T* is the temperature variation. The temperature sensor was measured in a temperature range of 25–50 °C, which covers the human skin temperature range.

(3) $$\Delta R=R-R_{0}$$

Figure 6A shows the normalized resistance change at 5 °C intervals ranging from 25 to 50 °C. The temperature sensor in this study exhibited excellent sensitivity of 1.114% °C^−1^ and a coefficient of determination of *R* ^2^ = 0.997, which shows good linearity. A higher sensitivity value indicates that the sensor is more responsive to different temperature variations. In our research, Ru‐ALD engineered DM‐V_2_CT_4_‐based temperature sensor showed much higher sensitivity than conventional metals such as platinum, nickel, and silver‐based temperature sensor, which reported the sensitivity as 0.32% °C^−1^, 0.48% °C^−1^, and 0.223% °C^−1^, respectively.^[^ ^14^ ^]^ Moreover, compared with pristine DM‐V_2_CT*_x_* MXene, which exhibited a sensitivity of 0.42% °C^−1^, the sensitivity increased 2.6 times higher after Ru‐ALD engineering (Figure S4, Supporting Information). Moreover, the sensor showed excellent reliability and stability when monitoring the resistance change during the heating and cooling processes. The long‐term measurement results were performed with and without enamel coating to further emphasize the durability of the MXene films by enamel encapsulation. As shown in Figure S6, Supporting Information, the temperature sensor fabricated with enamel coating demonstrated a stable output measurement, which indicates the durability of the MXene films by this encapsulation design. The sensor only showed a negligible resistance change of a maximum of 0.88% in the same temperature and experimental condition.

An efficient temperature sensor function and operation depend on the implemented materials' heat transport and the medium. In our case, we are dealing with a novel 2D layered V‐MXene with one carbide core sandwiched between two vanadium layers with terminal surface functional groups, as shown in Figure 1. The thermal and electrical characteristics of the medium mostly sensitize the V‐MXene's transport properties. The intrinsic thermal conductivity of V‐MXene facilitates in exchange of energy among various lattice vibrational phonon modes and hence the charge carriers.^[^ ^3^, ^4^ ^]^ On the other hand, V‐MXene's intrinsic electronic conductivity will drift the already generated carriers to the terminal electrodes and connecting wires.^[^ ^4a–d^ ^]^ Generally, fast traversing phonons majorly carry the heat in 2D nanomaterials.^[^ ^2^, ^3^, ^4^ ^]^ It is expected from the carbide core in V‐MXene must have sp^2^/sp^3^ carbon atoms and some lattice or graphitic defects among those layers throughout the MXene structures. Hence, those structural defects among the MXene layers lead to a considerable amount of phonon scattering during heat transport and are influenced by the MXene's surface functional groups.^[^ ^4c,e^ ^]^ As long as the sensitivity of the MXene materials is a concern, its phonon scattering and the presence of layers defects could be susceptible to any surface modification, such as by Ru‐ALD. Similar behaviors are also expected and reported in many graphene‐like materials.^[^ ^15^ ^]^

During V‐MXene's thermal conduction, the phonons could scatter with other internal phonons, associated graphitic/layered defects, and their interfaces, causing an electronic transport in an extended conduction band state. Each defect center or site may act as a trapping or de‐trapping charge center, causing electron mobility. Depending upon the availability of defect sites and density, the electron transit or drifting will be slower or faster, linking to the thermal vibration energies. While Ru‐ALD is implemented over the V‐MXene surface, its overall thermal carrier transport phenomena are significantly enhanced through the modified electronic conductivity and work function. On heating the sensor device/electrode, the Ru metal atoms/clusters inject excessive electron carriers into the lattices of layered V‐MXene materials. During these e^−^ injection processes, multiple phonon scattering occurs at various scattering centers or agents, for example, defect centers or the interface of Ru/V‐MXene. The possible presence of defects or trapping sites in the multilayered 2D V‐Mxene structures could strongly induce charge carrier densities. Considering the decrease in generating or releasing charge carriers due to the availability of various trapping sites, the defect energy state can be assigned just below the conduction band (Figure 6D). The trapping and de‐trapping processes at scattering centers result in e^˗^‐h+ pair recombinational losses, as discussed in the above PL spectra. A delayed recombination process is expected due to the multiple scattering events, such as electron‐electron/phonon and their respective defects and grain boundaries.

The V‐MXene's flake‐like morphology and layered structure support the hopping charge transport and conduction model, like graphene‐like materials. However, in V‐MXene, the inter‐flake charge transport mediated by tunneling transport is further enhanced by the Ru atoms/clusters, whereas intra‐flakes charge transport/conduction is facilitated by hopping.^[^ ^3^, ^13^, ^15^ ^]^ During thermal conduction, the generated phonon could interact or scatter with the conduction electrons, defects sites, and other associated phonons, hence undergoing multiple scattering, causing diffusive charge carriers. Especially in 2D graphene‐like MXene structure, the charge transport within an individual V‐MXene flake could be hopping type due to their intrinsic electronic structure and their delocalized defect states below the conduction band. Therefore, it is expected that the charge transport within the V‐MXene flake (intra‐flake transport) may be hopping type, whereas among the flakes (inter‐flake transport) can be tunnel type, as depicted in Figure 6D. While heating during the sensing measurement, the Ru‐atoms/clusters possibly inject excess charge carriers, that is, electrons, to the multilayered V‐MXene. During these excess charge carriers' injection processes, multiple scattering happens due to the presence of several trapping sites or defect centers at the multilayered V‐MXene structure leading electron drifting process.

Figure 6B,C show examples of the temperature sensor, which can detect the temperature difference directly. When cold and warm water was dropped on the sensor, which was encapsulated with enamel coating, the sensor responded instantly to the temperature difference caused by the abrupt drop of water (Figure 6B). The normalized resistance change was 2.96% and −6.39% for the cold and warm water (Figure S7, Supporting Information), which indicates the temperature of 27.2 ° and 19.2 °C, respectively. To demonstrate the sensor response to the continuous change of external temperature, the sensor was attached to a glass beaker to detect the temperature of the containing water. Warm, ice and hot water were sequentially added to the beaker, continuously changing the beaker's temperature. As shown in Figure 6C, the corresponding temperature can be calculated from the resistance variation of the sensor. An infrared camera was used to record the temperature variation of the beaker simultaneously during the entire experiment, as shown in the inset image.

### 2.5 Applications for Human Healthcare Monitoring and Human–Machine Interface

**Figure 7:**
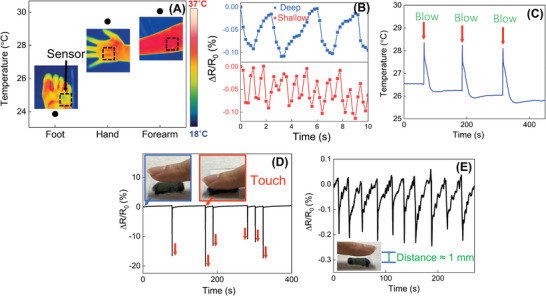
Ru‐ALD engineered DM‐V_2_CT*_X_* MXene real‐time skin temperature, breathing, blowing, touching, and proximity detection. A) Practical human skin temperature sensing measurements and their corresponding infrared images. B) Deep breathing (top blue color) and rapid/shallow breathing (bottom orange color). C) Blowing test. D) Contact touching of the fingertip. E) Proximity noncontact touch detection.

This temperature sensor has great potential to be used in various applications related to our daily‐life scenarios, such as healthcare, electronic skins, and human–machine interface. This study demonstrated several applications of their excellent sensitivity, linearity, and reliability (**Figure** **7**).

Human body temperature provides important aspects of health and physiology. For example, as the heat generates from the center of the body and spreads throughout the protruding body parts such as the nose, ears, and toes through blood circulation, the temperatures of these parts are relatively low. Due to the small size of the sensor and the fact that it can be attached to different parts of the human body, it can detect various human skin temperatures. As Figure 7A demonstrates, the temperature sensor was attached to the foot (toes), hand (palm), and forearm, measuring the temperature of 23.9 °C, 29.4 °C, and 30.1 °C, respectively. This measurement of skin temperature exhibited the skin temperature deviation of different body parts. For instance, the foot's temperature was relatively low compared to the other two parts, and the coolness can indicate a sign of poor circulation among the other extremities.

Furthermore, respiration is a vital human physiological signal closely related to human life and activity. Figure 7B,C demonstrate applications related to breathing and blowing monitoring. For breathing monitoring, the temperature sensor was placed in front of the nostrils of a volunteer. Since the temperature of the human exhalation is higher than that of the inhalation, a decrease and increase in normalized resistance change clearly occurred as breathing was repeated (Figure 7B). Additionally, as the measured resistance rose and fell periodically, we can distinguish the shallow (blue) and rapid (red) breathing appearance. For example, it indicates that the breathing rate of volunteers was 4 and 12 times within 10 s when shallow and rapid breathing was carried out, respectively. Moreover, as shown in Figure 7C, when the top surface of the encapsulated temperature sensor was blown, the ambient air temperature changed between 26 and 28 °C as the temperature increased due to warm air blowing. Notably, the lower temperature value after the blowing sequence was due to the endothermic process when the evaporation of a thin layer of fog, which was formed when warm air was blown, was accompanied.

Figure 7D shows the clear responses of touch detection which was initiated by the direct contact of the finger on the sensor. The normalized resistance variation showed a negative value ranging from −10% to −20% since the resistance decreased sharply when the fingertip, which had a relatively higher temperature, approached the sensor. Moreover, the cross‐check of the effect of the pressure of the sensor was introduced by comparing the normalized resistance change when touching the sensor with a wood stick (blue arrow), which doesn't have any heat source (Figure S8, Supporting Information). The decrease of the resistance due to the touch of a wood stick was negligible compared to the fingertip. This result indicates that our Ru‐ALD‐engineered DM‐V_2_CT*_X_* MXene‐based temperature sensor can selectively detect temperature variations without the interference of pressure caused by direct contact. In addition, as shown in Figure 7E, proximity noncontact touch detection was also demonstrated with a normalized resistance change of 0.1% since the fingertip acts as a heat source. This resistance change response could be effectively clarified, revealing the sensor's high sensitivity. These applications suggest our temperature sensor has the potential to be used in the human–machine interface, such as electronic skin or touch panel.

## 3 Conclusion

We successfully established a Ru‐ALD‐engineered DM‐V_2_CT*_x_*‐based temperature sensor with an excellent sensitivity of 1.11% °C^−1^ with good linearity performance. The developed sensor device is more responsive to different extreme temperature variations environments with excellent reliability and stability. The developed temperature sensor was also applied practically to measure the temperature change in various human organs. The non‐touch proximity sensing was also successfully recorded along with the respiration monitoring. The Ru‐ALD engineered V‐MXene sensor device has a 300% higher sensitivity performance than only V‐MXene‐based sensors credited to the V‐MXne's unique negative temperature coefficient, thermo‐electrical properties, and Ru atom/clusters doping effect mediated by tunneling transport in the inter‐flakes charge carriers. The V‐MXene and its Ru‐ALD process engineering have been used to develop a sensing device for the first time, where the ALD technique could offer a reliable and scalable device fabrication. A few‐layered delaminated V‐MXene was also successfully produced with a mild in situ etching technique to protect the environment without sacrificing scalability. This novel V‐MXene‐based sensor electrode and its precise, controlled modification by ALD could motivate to development of flexible electronic textile materials for mobile healthcare and contactless human–machine interface application.

## 4 Experimental Section

### Materials

Lithium fluoride (LiF), hydrochloric (HCl), hydrofluoric (HF), acids, and a quaternary ammonium salt, that is, tetramethylammonium hydroxide (TMAOH), were purchased from Sigma‐Aldrich and used as received without any further purification for the various MXene etching and processing. All the used solvents were of analytical grade, and the experiments were conducted in ambient environments unless specified by following proper safety standards and laboratory protocols.

### Few‐Layered Delaminated V_2_CT*_X_* MXene (DM‐V_2_CT*_X_*) Synthesis and Safety Protocol

Hydrofluoric acid (HF) is one of the primary etching components to prepare the MXene phase from its corresponding MAX phase. Since the Ti_3_C_2_T*_x_* (Ti‐MXene) discovery,^[^ ^1^ ^]^ HF has been used as one of the significant etchants for wet chemistry. However, HF in any concentration is toxic for direct contact with humans, for example, it causes severe burn and damage to the tissue due to the fast reactive hydrogen (hydronium ions) and the release of fluoride ions.^[^ ^16^ ^]^ Also, the HF's direct harsh chemical nature produces low‐quality, defective, unwanted multilayered bulk MXenes.^[^ ^17^ ^]^ Considering HF's associated human life risk and environmental contamination, it was imperative to introduce a milder chemical etching condition for MXene materials' sustainability and scalability. A milder etching condition using an optimized ratio of both HCl and LiF to carry out the in situ etching from the V_2_AlC MAX (V‐MAX) phase to the V_2_CT*_x_* MXene (V‐MXene) phase, as shown in Figure 1, was tried to introduce. It showed systematically and schematically the different stages involve in V‐MXene synthesis, from mild in situ etching to few‐layered V‐MXene through the delamination process. As a safety standard and protocol during the MAX phase etching process, high‐density polyethylene bottles were tried to use, where the etching process undergoes 90 h continuously under stir in a well‐conditioned fume hood. It was also ensured that the entire etchant and the V‐MAX phase in the polyethylene bottles should not exceed one‐third of its total used volume. Importantly, to avoid any sudden exothermic reactions, the ice bath was used around the polyethylene bottle where the etching of V‐MAX was going at ≈200 rpm stirring speed. It was also important to ensure while adding the V‐MAX phase to the etchant solution. It should be added slowly and completed in 5≈7 min per gram to avoid severe/rigorous reactions. After this stage, ≈15 min, the stirring speed must be increased slowly to 700 rpm and continued for 90 h. Once the etching was completed after 90 h, the subsequent essential stage of dilution and cleaning starts through the centrifuge. After many trials, the centrifuge speed was optimized to ≈ 4000 rpm for 8≈10 min to observe the sedimentation of the multilayered V‐MXene phase and a green supernatant. The green supernatant may be due to the residual vanadium metals or a small fraction of dissolved V‐MAX phase along with small intermetallics; hence can be decanted as waste. The dilution and centrifugation continued till the pH was above 6, for example, in the optimized cleaning case, and ≈2 L of DI water per ≈1 g V‐MAX phase to V‐MXene conversion was used.

In the second delamination stage, as per Figure 1, the as‐synthesized wet multilayered V‐MXene was immediately added to 10 wt% TMAOH solution in water, stirring at 700 rpm for 12 h continuously at room temperature in a fumehood. The idea of using the liquid stage wet multilayered V‐MXene instead of dring was to efficiently use already pre‐intercalated molecules among the V‐MXene layers for better mobility of both TMA^+^ and OH^−^ ions so that the possibility of producing delaminated, few‐layered V‐MXene would be higher. After completing the delamination process by large organic TMAOH molecules for 12 h, it was subjected to the 2000 rpm centrifuge and cleaning process till the supernatant solution became nearly colorless. However, the centrifuge tube's hand shaking or a few minutes of the sonication during the centrifugation/decantation process was crucial in safeguarding the sediment was well dispersed in every fresh batch of DI water. Post‐delamination process, delaminated V_2_CT*_X_* MXene (DM‐V_2_CT*_x_*) was used for the ALD process as follows.

### Ruthenium Atomic Layer Deposition Process on DM‐V_2_CT*_x_*

A traveling‐wave type thermal ALD reactor (Lucida D‐100, NCD technology, Korea) was used to grow Ru films on SiO2/Si wafers and DM‐V_2_CT*_x_* for material characterization and targeted sensing electrodes, respectively. For metal Ru film deposition, a Ru metal‐organic precursor, (tricarbonyl(trimethylenemethane)ruthenium, Ru(TMM)(CO)_3_) provided by TANAKA Precious Metals (Japan), and O_2_ as reactant used in the study. The following experimental procedure was used for the Ru films deposition to ensure the self‐limiting film growth and linear growth with ALD cycles from the earlier reported: 10 s pulsing of the Ru(TMM)(CO)_3_, 10 s of N_2_ purging, 10 s pulsing of the O_2_ reactant gas, and 10s of N_2_ gas purging. Repeating ALD cycles resulted in a film with the desired thickness by repeating one ALD cycle. A carrier gas of high‐purity N_2_ gas (99.999%) was supplied at a flow rate of 100 standard cubic centimeters per minute (sccm) to facilitate the transfer of the precursor to the chamber. It was ensured that the by‐products of the reaction and the excess chemical were removed from the reaction chamber by a purging time of 10 s between the successive precursor and reactant doses. The precursor was vaporized in a bubbler cooled to 10 °C to obtain a vapor pressure of ≈0.4 Torr and carried by N_2_ gas at a flow rate of 100 sccm. Further, the delivery lines for precursors and reactants were kept at 100 °C during the reaction to prevent condensation. This novel Ru‐precursor had a simple and small molecular structure that weakened the intermolecular forces, resulting in high vapor pressure, demonstrating a superior Ru‐ALD process to use the precious Ru metal on V‐MXene efficiently. By performing the deposition at 220 °C under the self‐limiting conditions, an ultralow amount of Ru was deposited onto the DM‐V_2_CT*_x_* substrate for the sensing and healthcare applications, as schematically shown in Figure 1. ALD directly offered a significant benefit over the cost of the processes because it reduced the amount of precious metals used and maximized their utilization. Moreover, ALD was an effective technique for dispersing active materials onto substrates with large surface areas, such as MXene, just performing a few ALD cycles in this current work.

### Advanced Physiochemical Characterizations

The as‐synthesized various MXene materials' crystallographic phases were analyzed by X‐ray diffractometer (XRD, PANalytical Xpert PRO MRD with Cu‐K*α* radiation with a wavelength (*λ*) of 1.54 Å). FESEM (HITACHI S‐4800) was used to investigate the overall morphology. The internal microstructure, exact 2D layered information, constituent elements, and stochiometry were probed by ultra‐high‐resolution transmission electron microscopy (UHR‐TEM, FEI Themis Z, Thermo Fisher Scientific, both high‐resolution TEM and STEM across the entire acceleration voltage range of 30 to 300 kV). This UHR‐TEM was equipped with next‐generation spherical aberration correction technology and fast, accurate elemental mapping quantification through its Super X or Dual X EDS detectors. The dual‐focused ion beam device was used to process the V‐MXene, delaminated V_2_CT*_x_* MXene (DM‐V_2_CT*_x_*), and Ru‐ALD engineered DM‐V_2_CT*_x_* (Ru@DM‐V_2_CT*_x_*) electrode samples for UHR‐TEM, which required high‐latency technology. The work function of sensor electrodes was evaluated by UPS and was collected by an AXIS Ultra DLD (Kratos, Inc.) equipped with a He I source (hv = 21.2 eV) at the Korea Basic Science Institute (KBSI).

### Preparation and Evaluation of Temperature Sensor

An electrode of the sensor was first formed by applying the silver paste on each end of V‐MXene coated on a substrate with a brush. The distance between the two electrodes was fixed at 1 cm. Subsequently, the sensor was encapsulated by enamel coating to ensure mechanical stability and prevent interference caused by humidity. All resistance data were acquired using a digital multi‐meter device and recorded with the laboratory virtual instrument engineering workbench. The temperature sensor performance measurements were carried out on the hot plate. In addition, the targets' temperature was simultaneously measured using an infrared thermal imaging camera (T560, FLIR) as a reference.

### Experiments on Human Subjects

The experiment for temperature sensors of skin temperature, breathing, and blowing tests were performed at the Pusan National University [institutional review board (IRB) approved protocol: 2018_86_HR].

## Conflict of Interest

The authors declare no conflict of interest.

## Supporting information

Supporting information

## Data Availability

The data that support the findings of this study are available in the supplementary material of this article.

## References

[1] M. Naguib , M. Kurtoglu , V. Presser , J. Lu , J. Niu , M. Heon , L. Hultman , Y. Gogotsi , M. W. Barsoum , Adv. Mater. 2011, 23, 4248. 21861270 10.1002/adma.201102306

[2] a) G. Deysher , C. E. Shuck , K. Hantanasirisakul , N. C. Frey , A. C. Foucher , K. Maleski , A. Sarycheva , V. B. Shenoy , E. A. Stach , B. Anasori , Y. Gogotsi , ACS Nano 2020, 14, 204; 31804797 10.1021/acsnano.9b07708

[3] a) A. VahidMohammadi , A. Hadjikhani , S. Shahbazmohamadi , M. Beidaghi , ACS Nano 2017, 11, 11135; 29039915 10.1021/acsnano.7b05350

[4] a) G. Ying , S. Kota , A. D. Dillon , A. T. Fafarman , M. W. Barsoum , FlatChem 2018, 8, 25;

[5] M. Saeidi‐Javash , Y. Du , M. Zeng , B. C. Wyatt , B. Zhang , N. Kempf , B. Anasori , Y. Zhang , ACS Appl. Electron. Mater. 2021, 3, 2341.

[6] R. Chen , T. Luo , D. Geng , Z. Shen , W. Zhou , Carbon 2022, 187, 35.

[7] a) X. Zhao , L.‐Y. Wang , C.‐Y. Tang , X.‐J. Zha , Y. Liu , B.‐H. Su , K. Ke , R.‐Y. Bao , M.‐B. Yang , W. Yang , ACS Nano 2020, 14, 8793; 32644797 10.1021/acsnano.0c03391

[8] M. Naguib , O. Mashtalir , M. R. Lukatskaya , B. Dyatkin , C. Zhang , V. Presser , Y. Gogotsi , M. W. Barsoum , Chem. Commun. 2014, 50, 7420. 10.1039/c4cc01646g 24821374

[9] M. Khazaei , A. Ranjbar , M. Ghorbani‐Asl , M. Arai , T. Sasaki , Y. Liang , S. Yunoki , Phys. Rev. B 2016, 93, 205125.

[10] a) Y. Kotsugi , S.‐M. Han , Y.‐H. Kim , T. Cheon , D. K. Nandi , R. Ramesh , N.‐K. Yu , K. Son , T. Tsugawa , S. Ohtake , R. Harada , Y.‐B. Park , B. Shong , S.‐H. Kim , Chem. Mater. 2021, 33, 5639;

[11] Y. Hernandez , V. Nicolosi , M. Lotya , F. M. Blighe , Z. Sun , S. De , I. T. McGovern , B. Holland , M. Byrne , Y. K. Gun'Ko , J. J. Boland , P. Niraj , G. Duesberg , S. Krishnamurthy , R. Goodhue , J. Hutchison , V. Scardaci , A. C. Ferrari , J. N. Coleman , Nat. Nanotechnol. 2008, 3, 563. 18772919 10.1038/nnano.2008.215

[12] M. Kim , K.‐J. Kim , S.‐J. Lee , H.‐M. Kim , S.‐Y. Cho , M.‐S. Kim , S.‐H. Kim , K.‐B. Kim , ACS Appl. Mater. Interfaces 2017, 9, 701. 27936584 10.1021/acsami.6b12622

[13] Abid, P. Sehrawat , S. S. Islam , P. Mishra , S. Ahmad , Sci. Rep. 2018, 8, 3537. 29476091 10.1038/s41598-018-21686-2 PMC5824820

[14] a) L. Gwo‐Bin , H. Fu‐Chun , L. Chia‐Yen , M. Jiun‐Jih , Acta Mech. Sin. 2004, 20, 140;

[15] a) C. Zhou , X. Zhang , H. Zhang , X. Duan , presented at 2019 IEEE SENSORS , Montreal, October 2019;

[16] a) J. J. Kirkpatrick , D. S. Enion , D. A. Burd , Burns 1995, 21, 483; 8540973 10.1016/0305-4179(95)93254-h

[17] X. Sang , Y. Xie , M.‐W. Lin , M. Alhabeb , K. L. Van Aken , Y. Gogotsi , P. R. C. Kent , K. Xiao , R. R. Unocic , ACS Nano 2016, 10, 9193. 27598326 10.1021/acsnano.6b05240

