# Supplementary material for: Process Controlled Ruthenium on 2D Engineered V‐MXene via Atomic Layer Deposition for Human Healthcare Monitoring
Source: Adv Sci (Weinh). 2023 Feb 22;10(12):2206355. doi: 10.1002/advs.202206355 (PMC10131817; doi:10.1002/advs.202206355)
Supplement: Supplementary file 1 — Supporting information [file ADVS-10-2206355-s001.pdf]

Supporting Information

Process Controlled Ruthenium on 2D Engineered V-MXene via Atomic Layer Deposition for Human Healthcare Monitoring

Debananda Mohapatra, Yujin Shin, Mohd Zahid Ansari, Youn-Hye Kim, Ye Jin Park, Taehoon Cheon, Haekyoung Kim*, Jung Woo Lee*, and Soo-Hyun Kim*

D. Mohapatra, M. Z. Ansari, Y.-H. Kim, Y. J. Park, H. Kim
School of Materials Science and Engineering
Yeungnam University
Gyeongsan, Gyeongbuk 38541, Republic of Korea
E-mail: hkkim@ynu.ac.kr

Y. Shin, J. W. Lee
Department of Materials Science and Engineering
Pusan National University
Geumjeong-gu, Busan 46241, Republic of Korea
E-mail: jungwoolee@pusan.ac.kr

T. Cheon
Center for Core Research Facilities, Daegu Gyeongbuk Institute of Science & Technology (DGIST), Sang-ri, Hyeonpung-myeon, Dalseong-gun, Daegu, 711-873, Republic Korea

S.-H. Kim
Graduate School of Semiconductor Materials and Devices Engineering, Ulsan National Institute of Science and Technology (UNIST), Ulju-gun, Ulsan, 44919, Republic of Korea
E-mail: soohyunsq@unist.ac.kr

Keywords: atomic layer deposition (ALD), V_2CT_X MXene, precious metals, human-machine interface, healthcare monitoring

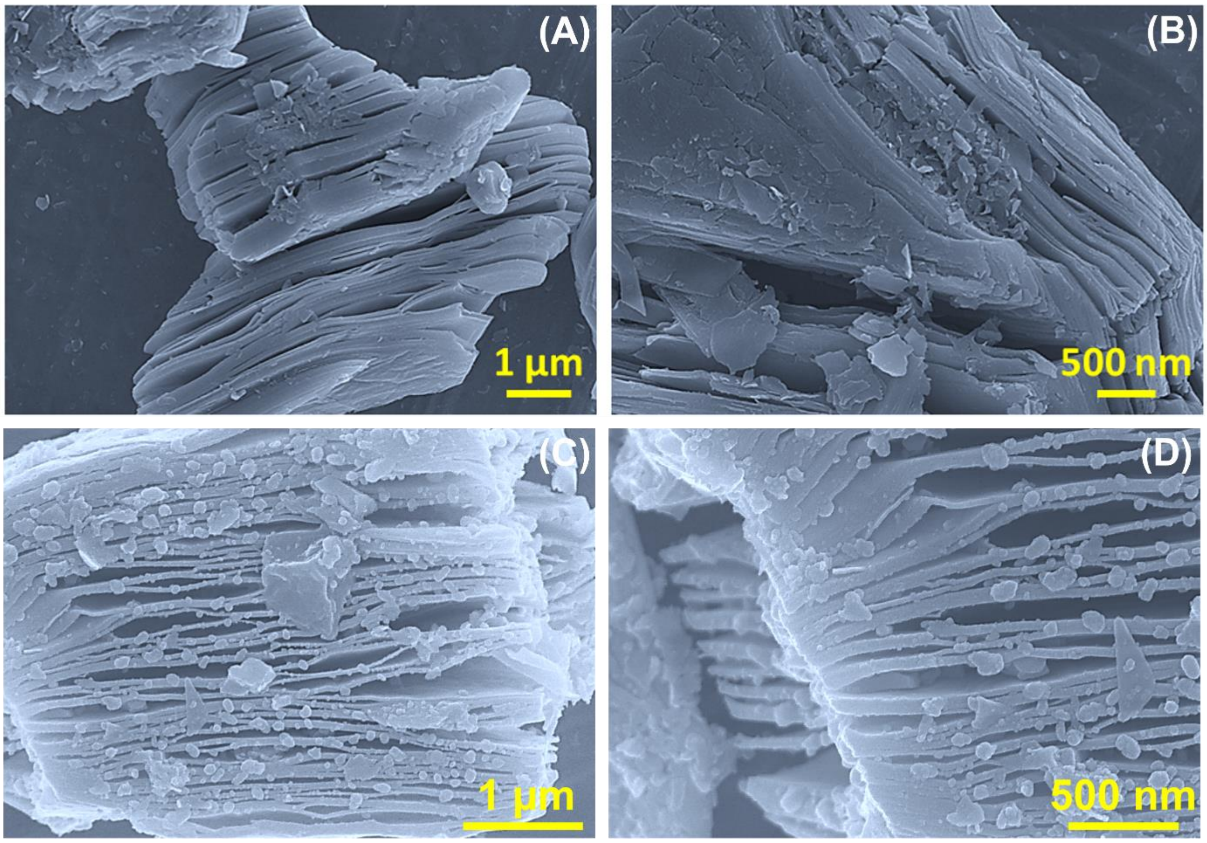

Figure S1. FE-SEM overall accordion-like open microstructure. (A, B) In-situ mild etched V_2CT_X (V-MXene) and (C, D) TMAOH intercalated delaminated V_2CT_X MXene (DM-V_2CT_X).

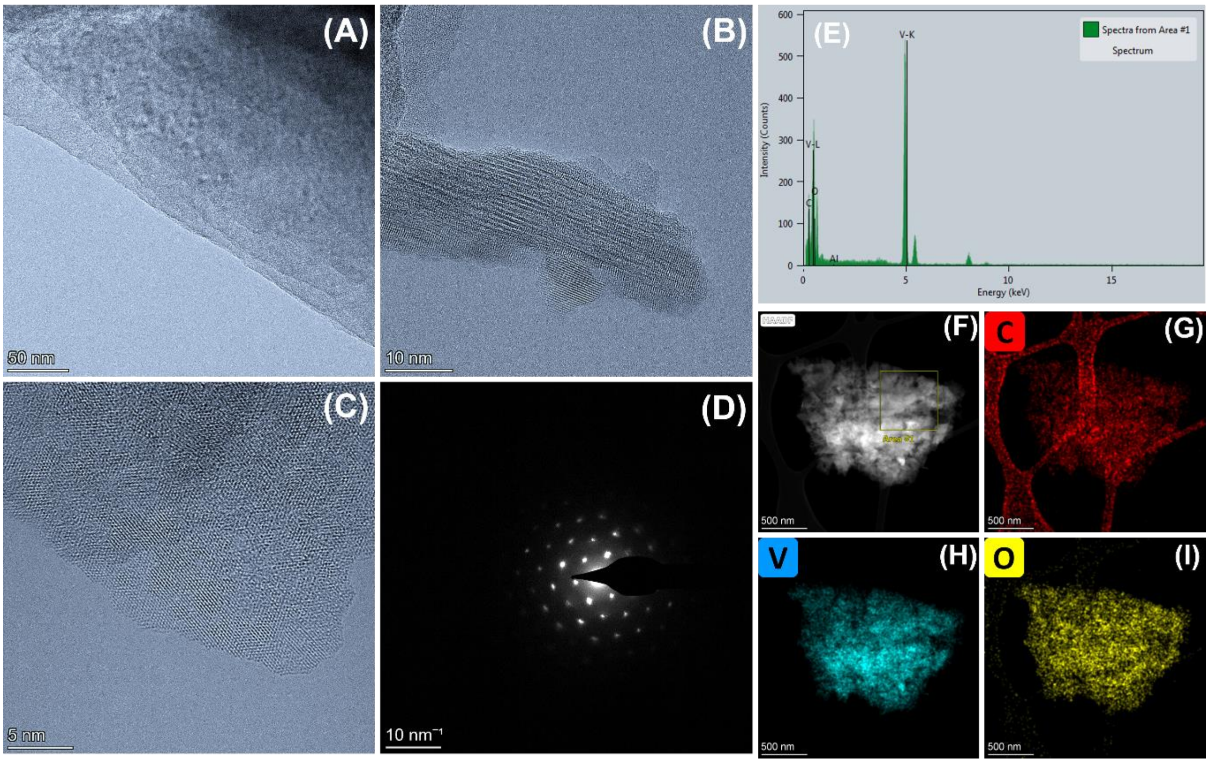

Figure S2. DM-V_2CT_X MXene microstructure, EDS, and elemental mapping. (A) TEM, (B, C) HR-TEM images, (D) SAED hexagonal lattice pattern, (E) EDS elemental spectra, and (F-I) elemental mapping images.

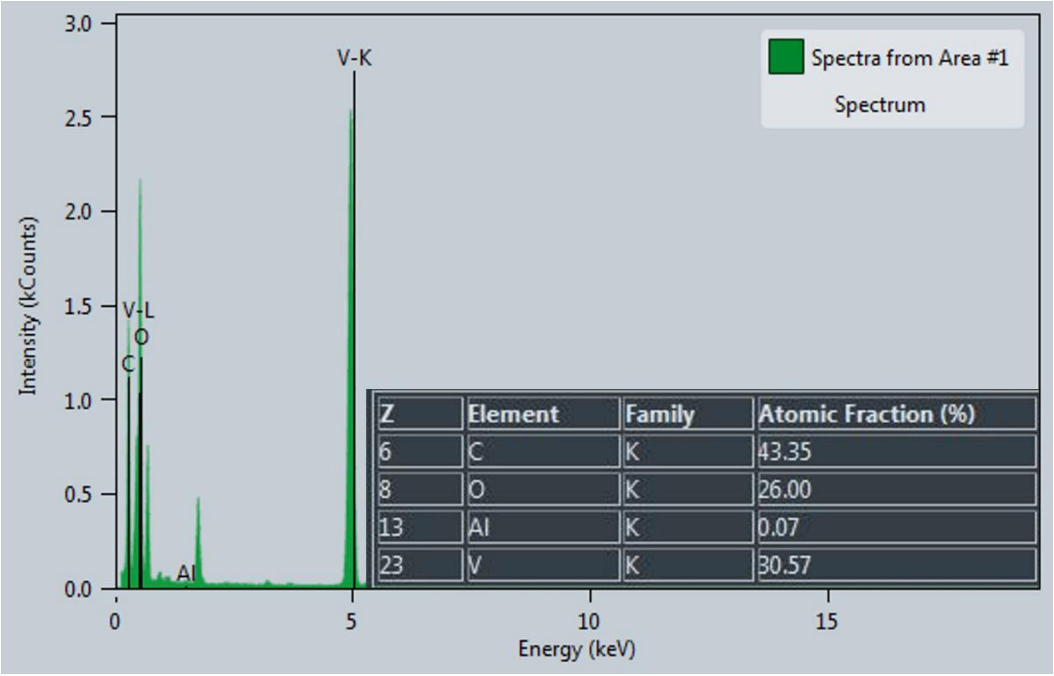

Figure S3. DM-V_2CT_X MXene microstructure's as-received EDS data and the statistic of each element to confirm the presence of both vanadium (V) and carbon (C) as major constituents.

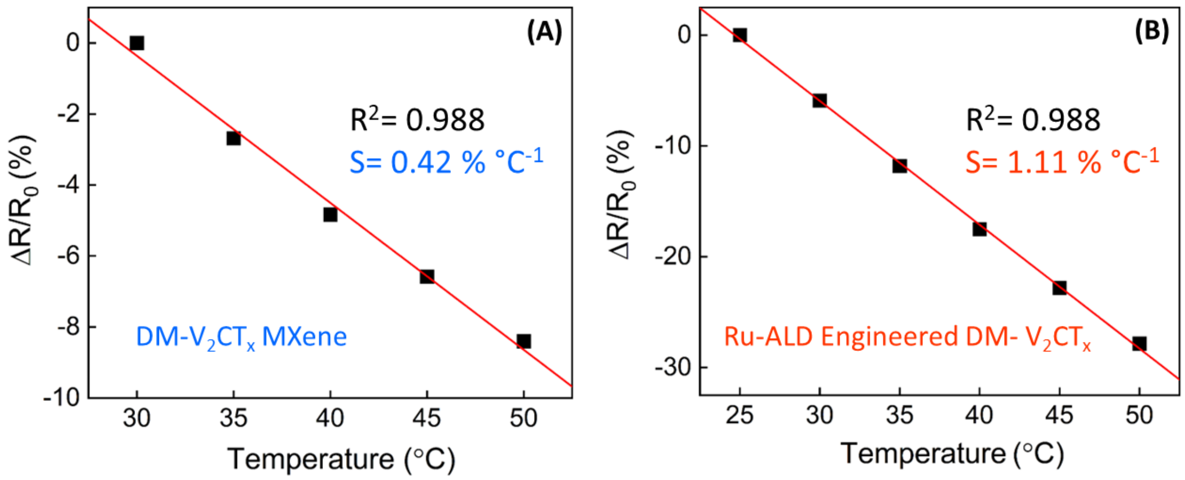

Figure S4. The temperature sensitivities of (A) DM-V_2CT_X MXene and (B) Ru-ALD engineered DM-V_2CT_X MXene.

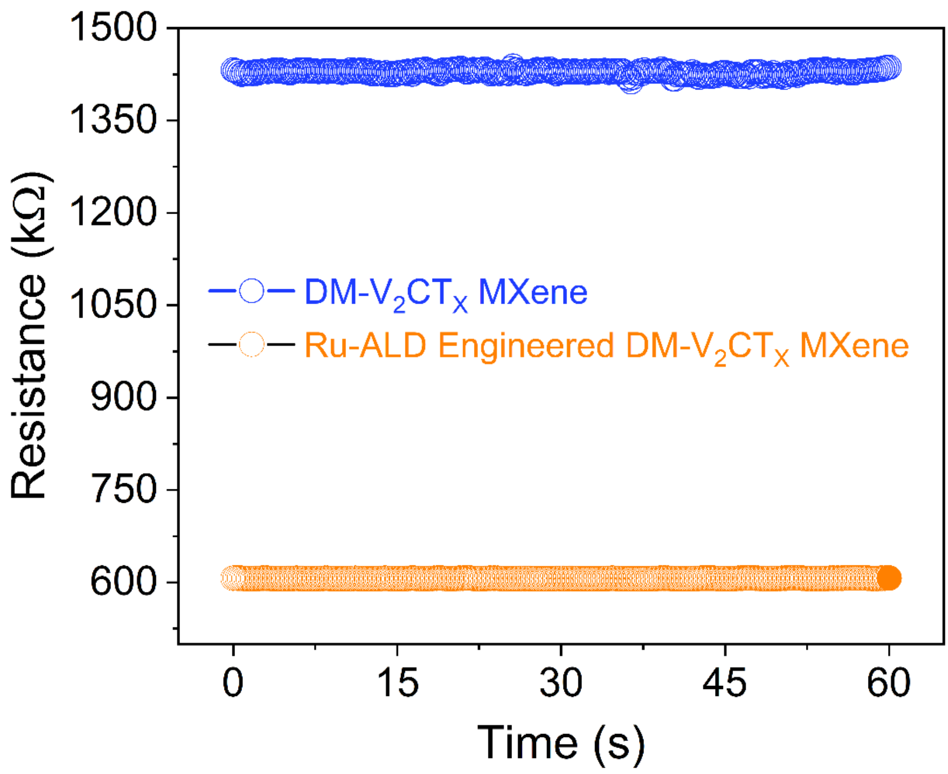

Figure S5. Change in resistance before and after Ru-ALD engineering to DM-V_2CT_X MXene.

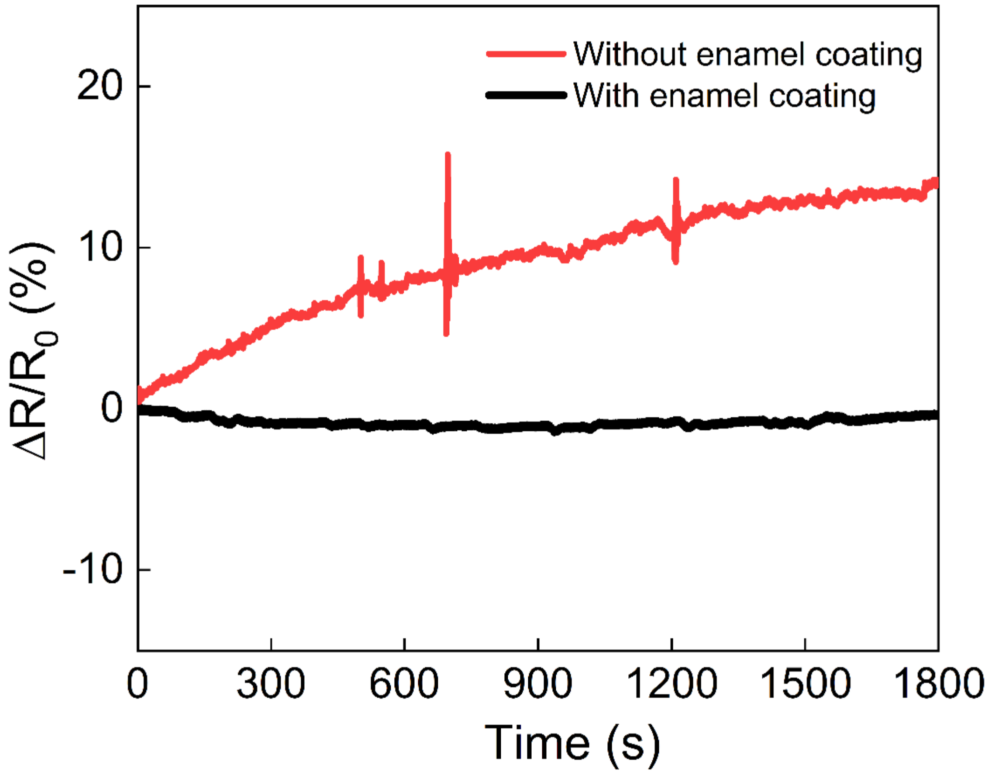

Figure S6. Durability of the MXene films by enamel encapsulation.

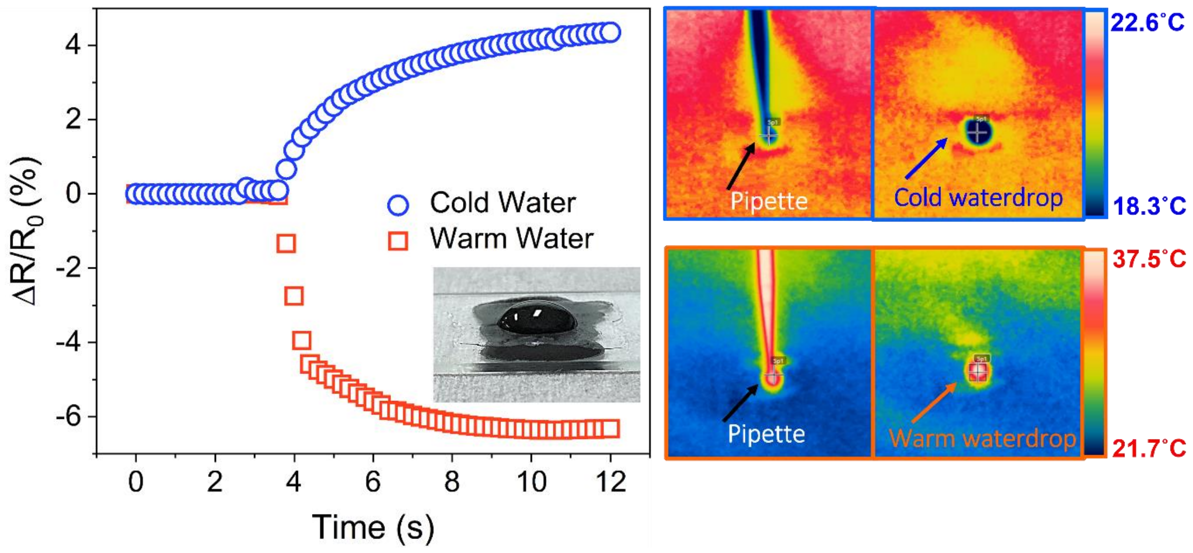

Figure S7. Ru-ALD engineered DM-V_2CT_X MXene dropping test (response time) – cold water (5˚C), warm water (40˚C) with infrared recordings.

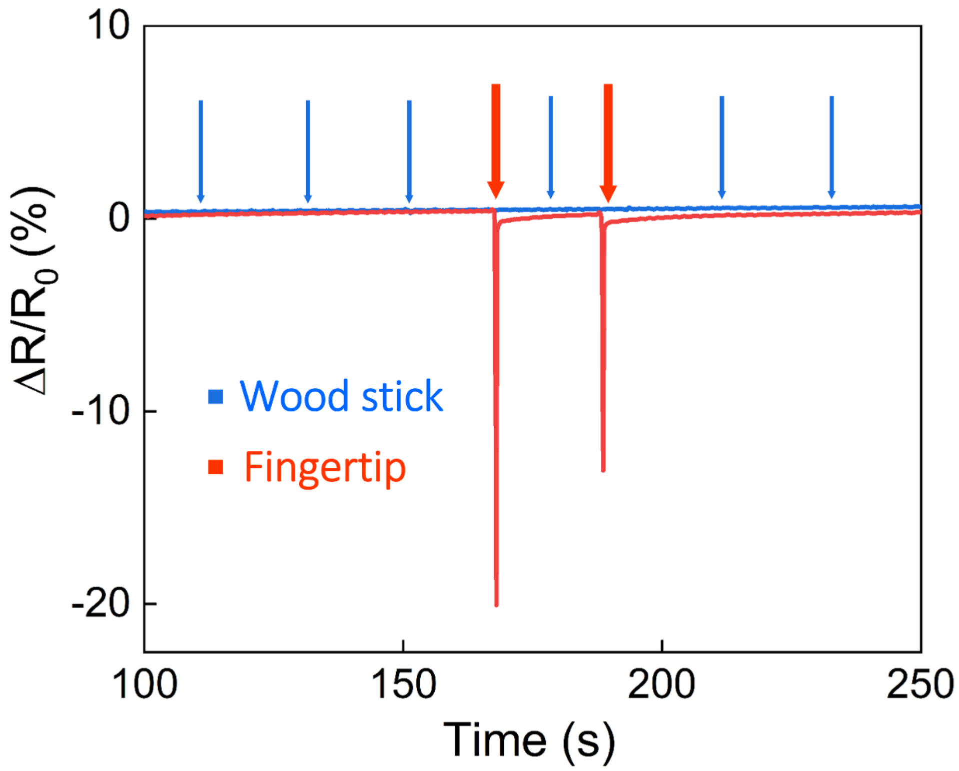

Figure S8. Cross-checking the effect of pressure or proximity sensing of Ru-ALD engineered DM-V_2CT_X MXene–Comparing fingertip & wood stick.
